# Identification of Genetic Modules Mediating the Jekyll and Hyde Interaction of *Dinoroseobacter shibae* with the Dinoflagellate *Prorocentrum minimum*

**DOI:** 10.3389/fmicb.2015.01262

**Published:** 2015-11-13

**Authors:** Hui Wang, Jürgen Tomasch, Victoria Michael, Sabin Bhuju, Michael Jarek, Jörn Petersen, Irene Wagner-Döbler

**Affiliations:** ^1^Helmholtz-Centre for Infection Research, Microbial CommunicationBraunschweig, Germany; ^2^German Collection of Microorganisms and Cell Cultures, Microbial Ecology and Diversity ResearchBraunschweig, Germany; ^3^Helmholtz-Centre for Infection Research, Genome AnalyticsBraunschweig, Germany

**Keywords:** *Roseobacter*, Dinoflagellate, pathogenicity, CtrA-phosphorelay, quorum sensing, symbiosis, type IV secretion system, plasmid

## Abstract

The co-cultivation of the alphaproteobacterium *Dinoroseobacter shibae* with the dinoflagellate *Prorocentrum minimum* is characterized by a mutualistic phase followed by a pathogenic phase in which the bacterium kills aging algae. Thus it resembles the “Jekyll-and-Hyde” interaction that has been proposed for other algae and *Roseobacter*. Here, we identified key genetic components of this interaction. Analysis of the transcriptome of *D. shibae* in co-culture with *P. minimum* revealed growth phase dependent changes in the expression of quorum sensing, the CtrA phosphorelay, and flagella biosynthesis genes. Deletion of the histidine kinase gene *cckA* which is part of the CtrA phosphorelay or the flagella genes *fliC* or *flgK* resulted in complete lack of growth stimulation of *P. minimum* in co-culture with the *D. shibae* mutants. By contrast, pathogenicity was entirely dependent on one of the extrachromosomal elements of *D. shibae*, the 191 kb plasmid. The data show that flagella and the CtrA phosphorelay are required for establishing mutualism and prove a cell density dependent killing effect of *D. shibae* on *P. minimum* which is mediated by an unknown factor encoded on the 191 kb plasmid.

## Introduction

Members of the *Roseobacter* clade within the Alphaproteobacteria often dominate bacterial communities associated to marine algae (Gonzalez et al., 2000; Riemann et al., 2000; Alavi et al., 2001; Allgaier et al., 2003; Tujula et al., 2010; Ankrah et al., 2014; Chen et al., 2015). Uncultivated lineages of the *Roseobacter* clade were shown to play different roles during successions induced by algal blooms in the North Sea (Teeling et al., 2012; Wemheuer et al., 2014; Voget et al., 2015). Interactions between roseobacters and marine algae can be mutualistic (Amin et al., 2015; Durham et al., 2015), pathogenic (Case et al., 2011; Fernandes et al., 2011; Paul and Pohnert, 2011; Gardiner et al., 2015), or shift between both (Seyedsayamdost et al., 2011b; Wang et al., 2014a).

The *Roseobacter* strain *Ruegeria* sp. R11 was shown to induce bleaching of the macroalgae *Delisea pulchra* at elevated temperatures and when biosynthesis of quorum sensing (QS) inhibiting furanones by the algae was impaired (Case et al., 2011). *Ruegeria* sp. TM1040 and the heterotrophic dinoflagellate *Pfiesteria piscicida* display a mutualistic interaction, showing bacterial uptake of the algal osmolyte dimethylsulfoniopropionate (DMSP; Miller and Belas, 2004) and microalgal uptake of growth promoting factors produced by the bacteria (Geng and Belas, 2010). Bacterial motility controlled by the cell cycle regulator CtrA and the histidine kinase CckA has been shown to be important in this interaction (Miller and Belas, 2006). Interestingly, a so-called *Roseobacter* Motility Inducer (RMI) extracted from cell-free supernatants of this bacterial species showed algicidal effect against *Tetraselmis striata* and *T. chuii* (Sule and Belas, 2013). *Ruegeria pomeroyi* produces algicidal lactones which show an inhibitory effect against the fresh water alga *Chlorella fusca* (Riclea et al., 2012). An algicidal compound termed roseobacticide is produced by *Phaeobacter inhibens* BS107 in the presence of *p*-coumaric acid, a degradation product of lignin released by aging coccolithophores like *Emiliania huxleyi* (Seyedsayamdost et al., 2011a,b). *P. inhibens* also produces tropodithietic acid (TDA), an antibacterial compound whose production is controlled by QS (Berger et al., 2011). Thus, the relationship between the coccolithophore *E. huxleyi* and *P. inhibens* might shift from a mutualistic stage, where the bacteria protect the algae from other bacteria through the synthesis of TDA, to a pathogenic stage where algal lysis is induced by the roseobacticides. For the biosynthesis of roseobacticides in *P. inhibens*, three precursor molecules are needed, namely phenylacetic acid, a plant growth promoter synthesized by the bacterium, *p*-coumaric acid derived from aging alga, and cysteine derived from the algal osmolyte DMSP which can be used as a carbon source by the bacterium. Strikingly, while each of these molecules is beneficial in the mutualistic phase, they might be combined to make toxins in the pathogenic phase (Seyedsayamdost et al., 2014).

Our work on the interaction between *Prorocentrum minimum*, a dinoflagellate that is capable of photoautotrophic growth but also feeds on algal prey upon phosphorous and nitrogen limitation (Stoecker et al., 1997; Johnson, 2015), and the *Roseobacter* species *Dinoroseobacter shibae* has demonstrated that indeed these two organisms switch from a mutualistic to an antagonistic stage in co-culture (Wang et al., 2014a), thus resembling the “Jekyll and Hyde” interaction proposed for *E. huxleyi* and *P. inhibens* (Seyedsayamdost et al., 2011b). *D. shibae* has been shown to provide vitamins B_12_ and B_7_ to its algal host (Wagner-Döbler et al., 2010). Here, we started to investigate the genetic mechanisms underlying both the mutualistic and the antagonistic relationship. *D. shibae* harbors a complex *N*-acyl homoserine lactone (AHL) mediated QS system regulating phenotypic traits that might be important for the interaction with dinoflagellates, e.g., flagella and two type IV secretion systems (T4SS). The T4SS are located on the 191 and 126 kb sister plasmids, respectively (Patzelt et al., 2013). Recently, we have demonstrated that the CtrA phosphorelay, an important regulatory circuit in Alphaproteobacteria (Brilli et al., 2010), is integrated into the QS system of *D. shibae* and controls flagella biosynthesis as well as the synthesis of AHLs with a C14 side chain (Wang et al., 2014b).

Here, we investigated the transcriptome of *D. shibae* in co-culture with *P. minimum*. Based on those findings we then studied how deletion mutants for key genes, e.g., the autoinducer synthase *luxI*_1_, the histidine kinase *cckA*, genes from the flagella gene clusters as well as the complete 191 kb plasmid, changed the mutualistic or pathogenic phase of the co-culture between *P. minimum* and *D. shibae.*

## Materials and Methods

### Cultivation of Algae and Bacteria

The axenic culture of *Prorocentrum minimum* CCMP 1329 used in this work was obtained from the Provasoli-Guillard National Center for Marine Algae and Microbiota (NCMA, formerly the Provasoli-Guillard National Center for Culture of Marine Phytoplankton, CCMP). CCMP 1329 was cultivated as previously described (Wang et al., 2014a). *D. shibae* DFL-12 strains (**Table 1**) were grown at 30°C and 160 rpm in a chemically defined sea water medium (SWM) supplemented with 5 mM succinate, prepared as described previously (Tomasch et al., 2011). The co-cultures of *P. minimum* with *D. shibae* strains were prepared as previously described (Wang et al., 2014a). In brief, bacterial cells where added up to a final density of 10^7^ cells/ml to the culture of *P. minimum* immediately after subculturing the dinoflagellate in fresh L1 medium lacking vitamin B_12_ with an initial density of approximately 2000 cells/ml. The co-culture was grown in 100 ml batches in 300 ml Erlenmeyer flasks at 22°C under a 12:12 h light-dark cycle with a light intensity of about 40 μmol photons m^-2^ s^-1^. Growth of algae and bacteria was followed by cell counting using a BD FACS Canto flow cytometer (BD Biosciences, San Jose, CA, USA), according to the methods described previously (Wang et al., 2014a). Strains of *D. shibae* used in this study are listed in **Table 1.** All cultivations were performed in triplicates.

**Table 1:** *Dinoroseobacter shibae* strains used in this study.

Strain	Description	Reference
DFL-12	Wild-type	Biebl et al., 2005
Δ*luxI_1_*	Δ*luxI_1_*:: Gm^r^	Patzelt et al., 2013
*ΔctrA*	*ΔctrA*:: Gm^r^	Wang et al., 2014b
*ΔcckA*	*ΔcckA*:: Gm^r^	Wang et al., 2014b
Δ191-kb	Plasmid-cured mutant	Ebert et al., 2013
*ΔfliC*	Transposon mutant	Ebert et al., 2013
*ΔflgK*	Transposon mutant	Ebert et al., 2013


### Sampling

All cultures were monitored for 36 days. Sampling for cell counting by flow cytometry was performed every 3 days for the first 24 days and then every 6 days till the end of the experiment. For transcriptome analysis of the *P. minimum* and *D. shibae* wild-type in co-culture, sampling was performed at day 18, day 24, and day 30. 50 ml of the cultures were pelleted at 5000 rpm for 10 min and transferred into 2 ml Eppendorf tubes. The cell pellets were then covered with 1 ml Trizol reagent (Ambion, Life Technologies, Carlsbad, CA, USA), snap frozen in liquid nitrogen and stored at -70°C until RNA isolation. Two of the three samples were further used for RNA-seq analysis.

### RNA Isolation and Depletion of Ribosomal RNA

The total RNA of *P. minimum* and *D. shibae* wild-type strain in co-culture was extracted according to (Wang et al., 2014a). PolyATract System IV (Promega, Madsion, WI, USA) was used for isolation of algal mRNA according to the manufacturer’s instructions. For isolation of the bacterial mRNA Ribo-Zero Magnetic Kit for both plant leaf and gram-negative bacteria (Epicentre, Madsion, WI, USA) was used according to the manufacturer’s instructions. The final purification of the eluted mRNA was performed using ethanol precipitation.

### RNA Sequencing and Data Analysis

The library was prepared from ribosomally depleted total RNA using Scriptseq v2 RNA-Seq Library Preparation Kit (Epicentre, Madsion, WI, USA) following the manufacturer’s protocol. Briefly, 50 ng of ribosomally depleted total RNA was chemically fragmented. Reverse transcription was performed using random hexamers with a 5′ tail containing a unique tagging sequence. After cDNA synthesis, the terminal tagging oligonucleotide (TTO) that blocked the 3′ end was annealed to the 3′ end of the cDNA. Following extension with a DNA polymerase, a second, unique tagging sequence (complementary to the TTO) was added to the 3′ end of the cDNA. Purification of cDNA was done using Agencourt AMPure purification. The resulting di-tagged cDNA was amplified by PCR using primers that anneal to the two different tagging sequences on the cDNA to generate the library. Library purification was done using AMPure purification.

Libraries were assessed using the Agilent 2100 Bioanalyzer (Agilent Technologies, Santa Clara, CA, USA). For sequencing equal volumes of libraries (12 pM) were multiplexed on a single lane. Cluster generation was performed with cBot (Illumina, San Diego, CA, USA) using TruSeq SR Cluster Kit v3-cBot-HS (Illumina, San Diego, CA, USA). Sequencing was done on the HiSeq 2500 (Illumina, San Diego, CA, USA) using TruSeq SBS Kit v3 – HS (Illumina, San Diego, CA, USA) for 50 cycles. Image analysis and base calling were performed using the Illumina pipeline v 1.8 (Illumina, San Diego, CA, USA).

The sequencing output (50 bp single end short reads) of the Genome Analyzer IIx was controlled for general quality features using the fastq-mcf tool of ea-utils (http://code.google.com/p/ea-utils) and was mapped against the genome sequence of *D. shibae* (NC_009952.1, NC_009955.1 NC_009956.1, NC_009957.1, NC_009958.1, and NC_009959.1) using BWA v 0.5.9-r16 (Li and Durbin, 2009). Statistical analysis of the mapped read counts was performed in the R environment using the package edgeR (Robinson et al., 2010). Gene set enrichment analysis for the cluster of orthologous (COG) group was performed based on the hypergeometric distribution function (phyper) in R. Raw and processed data are available from the gene expression omnibus database under accession number GSE55371.

## Results

### Transcriptome Changes of *D. shibae* during Growth in Co-culture with *P. minimum*

**FIGURE 1:**
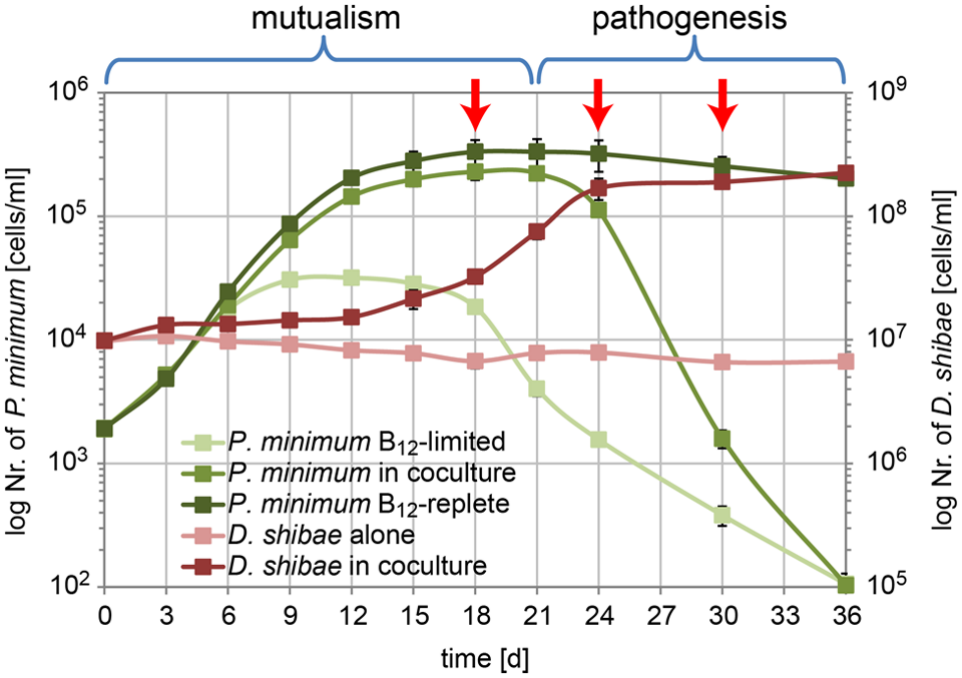
**Growth of *P. minimum* and *D. shibae* in co-culture.** In the mutualistic phase (day 0–day 21) both organisms profit from the co-cultivation in a medium where the dinoflagellate and the bacterium are not able to grow in single culture due to lack of vitamin B_12_ and organic carbon, respectively. In the pathogenic phase (day 21–day 36) the algae are killed by the bacteria, which continue to grow. Red arrows indicate the sampling time points for transcriptome analysis (modified from Wang et al., 2014a).

In our previous work we demonstrated that population dynamics of *P. minimum* in the co-culture with *D. shibae* exhibited a mutualistic phase (day 1 to day 21) where both partners profit from each other and a pathogenic phase (day 21 to day 36) where *D. shibae* kills aging dinoflagellate cells (Wang et al., 2014a). In the same study we analyzed the influence of light on the transcriptome of *D. shibae* at an early stage (day 12) of the co-cultivation (Wang et al., 2014a). Here, we used RNA-sequencing of samples from the same co-cultivation experiment to analyze the transcriptome of *D. shibae* at three different growth stages: (1) day 18 when *D. shibae* was in the mid-exponential growth phase, while *P. minimum* had entered the stationary phase; (2) day 24 when *D. shibae* had entered the stationary phase, while *P. minimum* started to decline; (3) day 30 when *P. minimum* had almost vanished, while *D. shibae* remained in the stationary phase (**Figure 1**). Two of the three biological replicate samples for each time point were used for RNA-seq analysis. The library sizes of the samples from different time-points increased with the increasing density of bacteria in the co-culture. Samples from day 18 reached only 15 and 139 k unique mapping reads, while samples from later time-points reached more than one to five million unique mapping reads (**Supplementary Table S1**). Despite these differences in sequencing depth the correlation between replicates was satisfactory for all time-points (**Supplementary Figure S1A**). To determine a cut-off for reliable changes in gene expression we reduced the sequencing depth for day 24 and day 30 to 100 k reads by resampling. The deviation in expression and log_2_ fold-change between the reduced and original dataset strongly increased for genes with an abundance of log_2_ cpm (counts per gene per million reads) lower than seven. We defined this value as the lower cut-off for reliable gene expression at day 18 (**Supplementary Figures S1B,C**). Only four of the 41 genes identified as significantly differentially expressed had a cpm-value slightly below this cut-off and therefor have to be treated with caution. This is consistent with our previous study, showing that small library sizes increase the error of the obtained reads per gene in particular for weakly expressed genes (Wang et al., 2014a).

**FIGURE 2:**
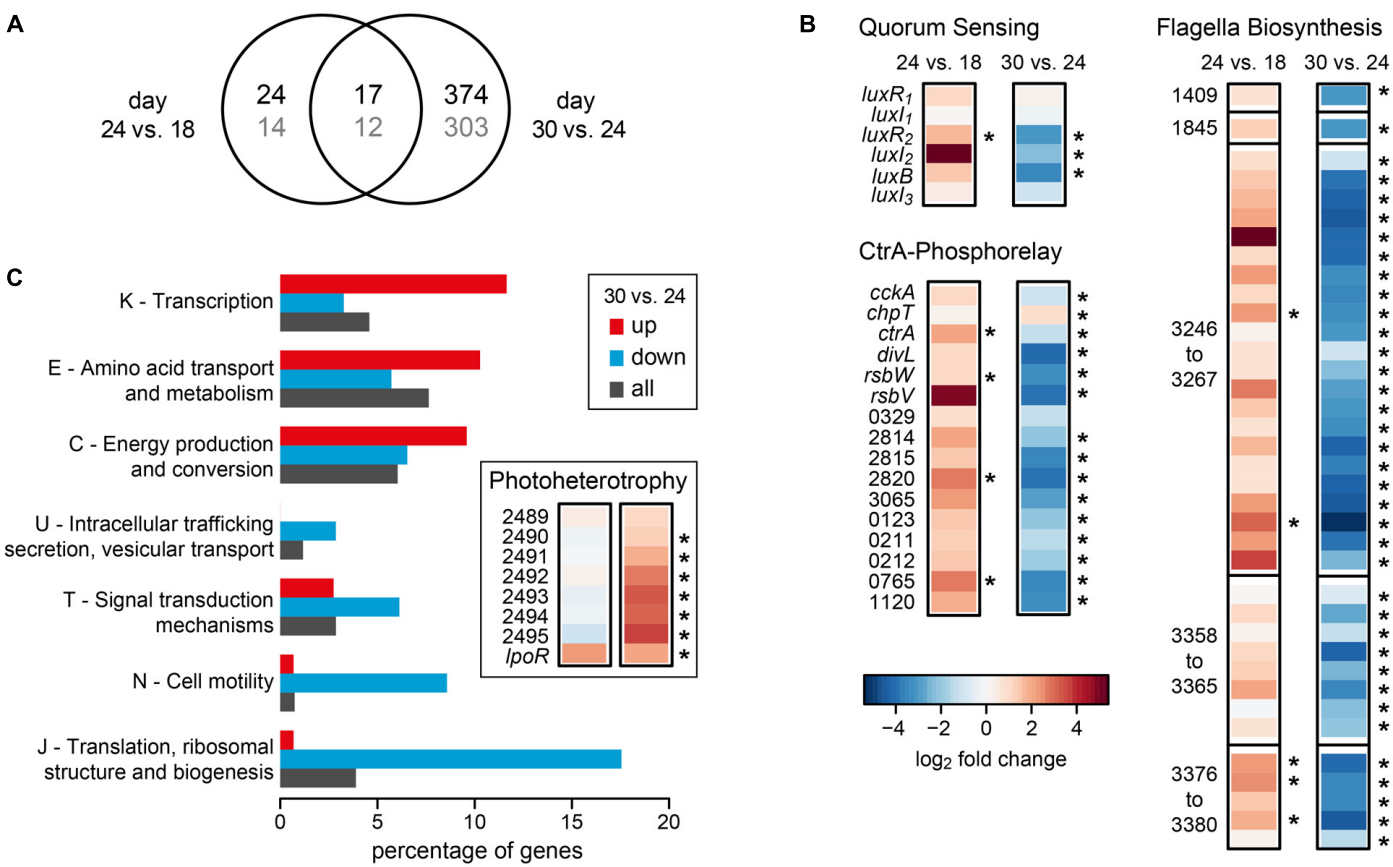
**Differential gene expression of *D. shibae* during the transition from mutualistic to pathogenic (day 24 vs. day 18) and from pathogenic to stationary phase (day 30 vs. day 24). (A)** Venn-diagram showing the overlap in the number of differentially expressed genes for both comparisons. The number of genes with COG-annotation is shown in gray. **(B)** Differential expression of genes of the Quorum sensing system, the CtrA phosphorelay and flagella biosynthesis (two single genes and three gene clusters). Asterisks indicate significant differences in expression (*p*-value < 0.05). Identifiers are either the gene symbols or the locus tags according to **Supplementary Table S2**. **(C)** Significantly enriched COG-categories (*p*-value < 0.1) for the comparison of day 30 with day 24. The percentage of genes of enriched categories is shown for up- and down-regulated genes. The percentage of all genes in the genome of *D. shibae* that have been assigned to the respective category is shown for comparison. Inlay: differential expression of genes with relevance for aerobic anoxygenic photosynthesis.

Comparing day 24 to day 18 we observed 41 of the 4192 protein-coding genes with a significant differential expression, 15 of them up- and 26 down-regulated. This number most likely underestimates the true number of differentially expressed genes because of the low sequencing depth at day 18. In contrast, we found 391 differentially expressed genes for the comparison of day 30 with day 24, 146 of them up- and 245 down-regulated. Both comparisons shared 17 differentially expressed genes (**Figure 2A**). The complete dataset can be found in **Supplementary Table S2**.

Interestingly, genes that had previously been identified as being part of the quorum-sensing controlled CtrA-phosphorelay (Wang et al., 2014b), were regulated in different directions during the two transitions analyzed here (**Figure 2B**). Specifically, when comparing the samples of day 24 to day 18, *ctrA* and its targets, the *luxR_2_I_2_* operon coding for an autoinducer synthase and the respective transcription factor of the QS system, the sigma-factor-antagonist *rbsW* and its repressor *rbsV* (Dshi_0072/73), genes controlling cyclic di-GMP, Dshi_0329/2814/2815/2820/3065 and other regulatory genes (Dshi_0123/0211/0212/0765) as well as the first gene of a Type II secretion locus (Dshi_1120) were consistently up-regulated. Moreover, the genes coding for components of the flagella, most of them located in three different clusters on the chromosome of *D. shibae* (Wang et al., 2014b), were all up-regulated.

When comparing day 30 (late pathogenic phase) to day 24 (early pathogenic phase), all of these genes were strongly and significantly down-regulated (**Figure 2B**). From the 391 differentially expressed genes found in this transition, 315 could be assigned to a COG category. **Figure 2C** shows that several COG categories were down-regulated, especially J (translation, ribosomal structure, and biogenesis), N (cell motility), T (signal transduction mechanisms), and U (intracellular trafficking, secretion, vesicular transport). By contrast, categories K (transcription), E (amino acid transport), and C (energy production) were higher expressed (**Figure 2C**). These data suggest that the cells had entered the stationary phase of growth at day 30. Since *D. shibae* is photoheterotrophic, and light has been shown to play an important role during adaptation to the stationary phase of growth (Soora et al., 2015), we had a closer look at genes important for photoheterotrophy (**Figure 2C** inset). Interestingly, two loci that have recently been associated with aerobic anoxygenic photosynthesis were up-regulated at this stage. The genes Dshi_2489-2595 might be involved in CO_2_ fixation and have previously been found to be permanently up-regulated in the light (Tomasch et al., 2011). The extrachromosomally located *lpor* gene codes for a functional light-dependent protochlorophyllide reductase of cyanobacterial origin (Kaschner et al., 2014). Its expression is controlled by light and nutrient limitation, and it is located on a 72 kb chromid which is indispensable for survival under starvation in the light (Soora et al., 2015). Thus, our data are in accordance with previous studies and suggest that *D. shibae* switches to a more photoheterotrophic life-style when substrates gained from the lysis of algae become scarce.

### Co-cultivation of *P. minimum* with *D. shibae* Knock-out Mutants

To determine how the different genetic modules shown to be expressed in a growth-phase dependent way in the co-culture of *D. shibae* with *P. minimum* influence mutualism or pathogenesis, we deleted key-genes and grew the respective mutants (**Table 1**) in co-culture with *P. minimum*. Deletion of the autoinducer synthase LuxI_1_ results in a QS null mutant which lacks morphological heterogeneity and down-regulated flagella synthesis and expression of the two T4SS (Patzelt et al., 2013). For the CtrA phosphorelay we studied deletions of the histidine kinase CckA (Δ*cckA*) and of the response regulator CrtA (Wang et al., 2014b).The Δ*ctrA* mutant described previously had by chance lost the 191 kb plasmid (Wang et al., 2014b) and is therefore designated Δ*ctrA*-191. Additionally we therefore investigated a mutant with intact *crtA* gene but lacking the 191 kb plasmid (designated Δ191; Ebert et al., 2013) that had been cured according to the strategy described (Petersen et al., 2012). For the components of the flagella, we used the structural genes *fliC* and *flgK* that had been deleted by transposon mutagenesis (Ebert et al., 2013). All of the strains were able to grow in single culture. Δ*luxI_1_* as well as Δ*cckA* and Δ*ctrA*-191 had a shorter lag phase and higher growth rate in artificial sea-water supplemented with succinate as c-source as reported before (Patzelt et al., 2013; Wang et al., 2014b). The same was true for both flagella mutants while the Δ191 mutant strain showed a prolonged lag-phase and lower growth-rate in single culture (**Supplementary Figure S2**).

**FIGURE 3:**
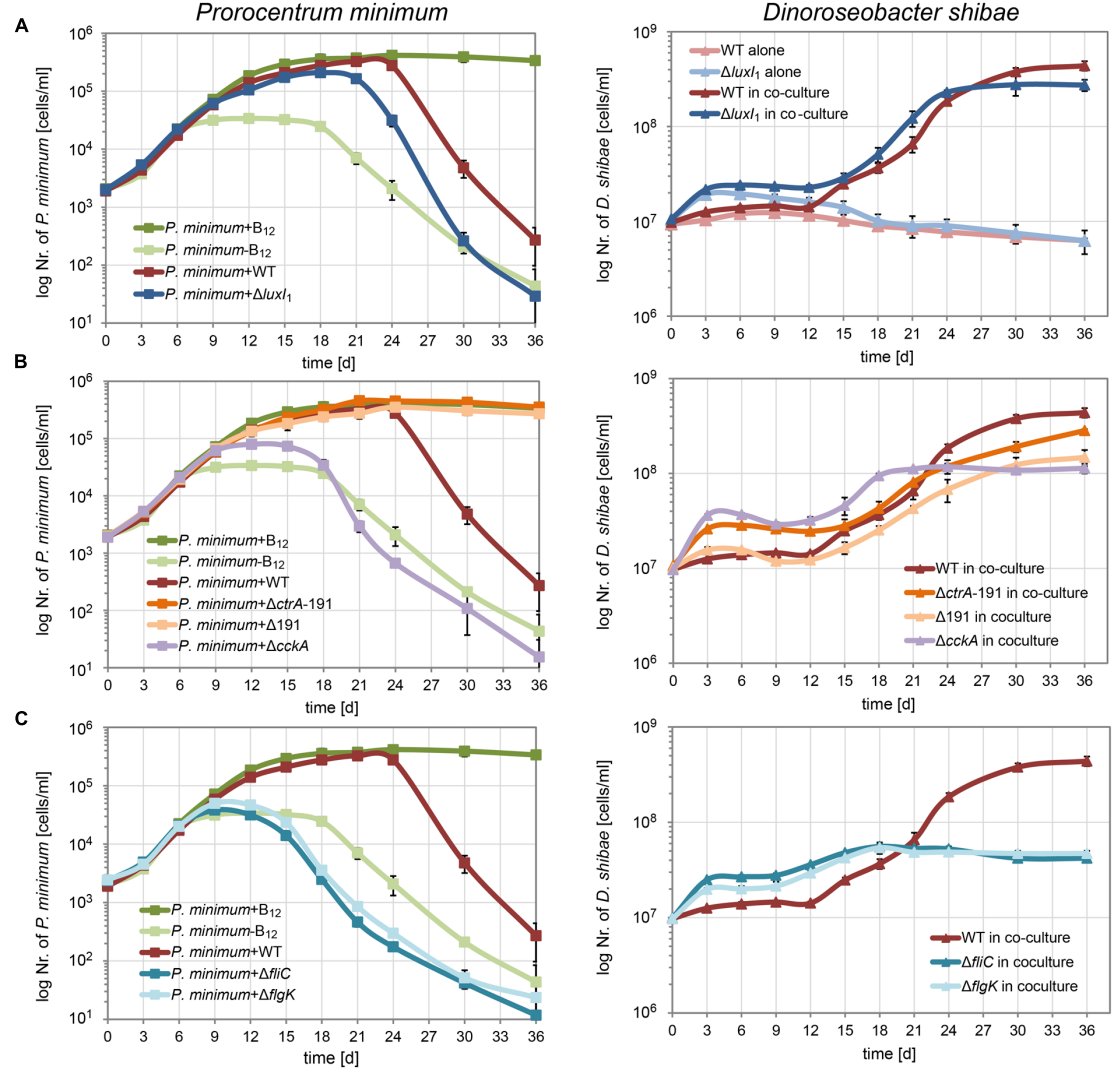
**Growth of *P. minimum* and different *D. shibae* mutants in co-culture.** *P. minimum* cell numbers are shown on the left, *D. shibae* cell numbers are shown on the right. The *P. minimum* positive and negative controls as well as the growth of the co-culture with wild-type *D. shibae* are shown in every panel for comparison. **(A)** Co-culture with the quorum sensing null-mutant *D. shibae* Δ*luxI_1_*, **(B)** with the phosphorelay knock-out mutants *D. shibae* Δ*cckA* and Δ*ctrA*-191, the latter additionally lacking the 191 kb plasmid, and a Δ191 kb cured mutant with intact *ctrA* gene, **(C)** with *D. shibae* Δ*fliC* and Δ*flik*, lacking functional flagella. Data represent mean and standard deviation of three biological replicates.

When the QS null mutant Δ*luxI_1_* was cultivated together with *P. minimum* (**Figure 3A**) it grew faster than the wild-type, in accordance with its smaller cell size and higher growth rate in pure culture described previously (Patzelt et al., 2013). In contrast to the growth of the wild-type, cell numbers of the QS null mutant doubled within the first 3 days, even when cultivated alone in the medium without C-source. In co-culture cell numbers doubled initially as well, but – like in the wild-type – substantial growth started only at day 12. From then on, the mutant grew marginally faster than the wild-type and it reached slightly lower final cell densities. Interestingly, the decrease in the cell numbers of *P. minimum* started 3 days earlier in co-culture with the QS null mutant than in co-culture with the wild-type. The earlier start of the pathogenic phase might be a result of the faster growth rate of the QS null mutant and indicates a dosage effect, i.e., a larger number of cells has a stronger killing effect, in accordance with previous observations (Wagner-Döbler et al., 2010).

Next, we tested how the CtrA phosphorelay affects the interaction with the dinoflagellate (**Figure 3B**). The Δ*cckA* knock-out strain, which lacks the histidine kinase required for phosphorylating CtrA, grew initially faster in co-culture than the wild-type but reached a lower final cell density, most likely because of lack of growth of the dinoflagellate. In this co-culture, the dinoflagellate showed only weak growth and started dying at day 18, comparable to the negative control. These data suggest that the CtrA phosphorelay is critically important for establishing the mutualistic symbiosis between *P. minimum* and *D. shibae*.

The two strains lacking the 191 kb plasmid, Δ*ctrA*-191 and Δ191, differed in their initial growth rates in co-culture, likely due to the presence or absence of the *ctrA* gene (**Figure 3B**). While Δ*ctrA*-191 grew faster than the wild-type, similar to the Δ*cckA* mutant, Δ191 grew just like the wild-type. However, both mutants completely lacked the ability to kill the dinoflagellate. These data prove that it is indeed an interaction with the bacteria that kills the algae. Moreover, they show that essential determinants of the pathogenicity of *D. shibae* toward the alga are located on the 191 kb plasmid.

Synthesis of flagella is controlled by CtrA in all Alphaproteobacteria studied so far (Greene et al., 2012). Furthermore, their role in attachment of *Ruegeria* sp. TM1040 to the surface of its dinoflagellate host has been demonstrated (Miller and Belas, 2006). Therefore we asked which role the flagella play for the interaction of *D. shibae* and *P. minimum*. We found that knockout of either the flagellin (*fliC*) or a flagella hook associated protein (*flgK*) led to reduced growth of the dinoflagellate in co-culture, comparable to the negative control (**Figure 3C**). This effect was even more pronounced than for the Δ*cckA* mutant (**Figure 3B**). The dinoflagellate already died at day 15, earlier than in the negative control, indicating that not only was the symbiosis not established, but the bacteria most likely actively killed cells of *P. minimum*. These data indicate that flagella have an important role in the interaction which is independent of the CtrA phosphorelay.

## Discussion

Here, we show that QS, CtrA phosphorelay and flagella biosynthesis are regulated in opposite directions in different phases of the co-culture between *D. shibae* and *P. minimum*: at the onset of the pathogenic phase, those three traits are up-regulated, while they are down-regulated in the late pathogenic phase which might resemble the stationary phase of growth for *D. shibae*. CtrA phosphorelay and flagella biosynthesis have been shown to be controlled by QS in *D. shibae* (Patzelt et al., 2013). Expression of QS controlled genes can be density dependent, depending on the structure of the regulatory network (Fuqua et al., 1994; Haseltine and Arnold, 2008) and therefore their observed up-regulation during growth of *D. shibae* with the dinoflagellate could be unrelated to the co-cultivation. However, we previously showed that the QS controlled genes are not expressed in a density-dependent way when *D. shibae* is cultivated alone in a defined medium (Patzelt et al., 2013). Therefore, the transcriptome data suggest a possible role of QS, CtrA and flagella in the interaction of *D. shibae* with the dinoflagellate.

The contribution of those traits toward mutualism or pathogenicity, respectively, was investigated by co-cultivating a number of different mutants of *D. shibae* with the dinoflagellate. We show that establishment of a mutualistic interaction required the CtrA phosphorelay and flagella. Loss of CckA or essential parts of the flagellum led to reduced growth stimulation of the dinoflagellate by the bacterium. The cell numbers did not exceed those of the negative control. Our data therefore confirm the important role of flagella for interaction with host organisms that has already been found for *Ruegeria* sp. TM1040 which actively swims toward algal products and uses flagella for attachment (Miller et al., 2004; Miller and Belas, 2006). In contrast to *D. shibae*, TM1040 does not possess a QS system. This demonstrates that the role of CtrA and its targets in co-culture is not necessarily dependent on cell–cell communication. Control of CtrA by QS similar to that of *D. shibae* has been found in the sponge-symbiont *Ruegeria* sp. KLh11 where QS activates flagellar motility while inhibiting biofilm formation and therefore might also be crucial for interaction with the host (Zan et al., 2012, 2013). Recently it has been demonstrated that nodule-specific cysteine-rich peptides produced by *Medicago* plant species induce the differentiation of intracellular *Sinorhizobium meliloti* cells into nitrogen-fixing bacteroids by disrupting the expression of cell-cycle regulators, among them CtrA, furthermore exemplifying its important role for bacterial interaction with host species (Penterman et al., 2014).

The most striking finding of our study is the essential role of the 191 kb plasmid for the pathogenicity of *D. shibae.* It is a “killer plasmid,” since strains cured from this plasmid have completely lost the ability to kill *P. minimum*. This plasmid harbors a T4SS that might be able to transmit virulence factors to the host cell (Petersen et al., 2012). However, plasmid-located T4SS are commonly associated with conjugational DNA transfer (Christie et al., 2005; Smillie et al., 2010). Since the 126 kb sister-plasmid that also carries a T4SS apparently cannot complement the loss of the 191 kb plasmid, it could be hypothesized that those two T4SS are functionally different. Both T4SS were expressed but not up-regulated in the pathogenic phase of the co-culture with the dinoflagellate (**Supplementary Table S2**). Alternatively, other genes that are present on the 191 kb plasmid could be mediating the observed killing effect.

## Conflict of Interest Statement

The authors declare that the research was conducted in the absence of any commercial or financial relationships that could be construed as a potential conflict of interest.
