# Supplementary material for: Identification of Genetic Modules Mediating the Jekyll and Hyde Interaction of Dinoroseobacter shibae with the Dinoflagellate Prorocentrum minimum
Source: Front Microbiol. 2015 Nov 13;6:1262. doi: 10.3389/fmicb.2015.01262 (PMC4643747; doi:10.3389/fmicb.2015.01262)
Supplement: Supplementary file 1 [file Table_1.PDF]

Supplementary Table S1

Identification of genetic modules mediating the Jekyll and Hyde interaction of *Dinoroseobacter shibae* with the dinoflagellate *Prorocentrum minimum*

Hui Wang[1§], Jürgen Tomasch[1§], Victoria Michael[2], Sabin Bhuju[3], Michael Jarek[3], Jörn Petersen[2] and Irene Wagner-Döbler[1#]

[§]contributed equally to this work

[#]corresponding author: Irene.Wagner-Doebler@helmholtz-hzi.de

[1]Helmholtz-Centre for Infection Research (HZI), Microbial Communication, Braunschweig, Germany

[2]German Collection of Microorganisms and Cell Cultures (DSMZ), Microbial Ecology and Diversity Research, Braunschweig, Germany

[3]Helmholtz-Centre for Infection Research (HZI), Genome Analytics, Braunschweig, Germany

Running title: Algae – bacteria interactions

Subject category: Microbe-microbe and microbe-host interactions

Supplementary Table S1. Mapping statistics of RNA-seq data.

No. of reads	Day 18a	Day 18b	Day 24a	Day 24b	Day 30a	Day 30b
Counted fragments	36,581	169,698	1,528,357	3,199,674	6,153,431	6,165,259
- uniquely	15,227	139,421	1,279,767	2,903,645	5,339,346	5,282,941
- non-specifically	21,354	30,277	248,590	296,029	814,085	882,318
Uncounted fragments	52,601,442	59,207,269	50,110,833	37,034,663	12,590,839	20,337,523
Total fragments	52,638,023	59,376,967	51,639,190	40,234,337	18,744,270	26,502,782
