# Supplementary material for: Identification of Genetic Modules Mediating the Jekyll and Hyde Interaction of Dinoroseobacter shibae with the Dinoflagellate Prorocentrum minimum
Source: Front Microbiol. 2015 Nov 13;6:1262. doi: 10.3389/fmicb.2015.01262 (PMC4643747; doi:10.3389/fmicb.2015.01262)
Supplement: Supplementary file 3 [file Image_1.PDF]

Supplementary Figure S1

Identification of genetic modules mediating the Jekyll and Hyde interaction of *Dinoroseobacter shibae* with the dinoflagellate *Prorocentrum minimum*

Hui Wang[1§], Jürgen Tomasch[1§], Victoria Michael[2], Sabin Bhuju[3], Michael Jarek[3], Jörn Petersen[2] and Irene Wagner-Döbler[1#]

[§]contributed equally to this work

[#]corresponding author: Irene.Wagner-Doebler@helmholtz-hzi.de

[1]Helmholtz-Centre for Infection Research (HZI), Microbial Communication, Braunschweig, Germany

[2]German Collection of Microorganisms and Cell Cultures (DSMZ), Microbial Ecology and Diversity Research, Braunschweig, Germany

[3]Helmholtz-Centre for Infection Research (HZI), Genome Analytics, Braunschweig, Germany

Running title: Algae – bacteria interactions

Subject category: Microbe-microbe and microbe-host interactions

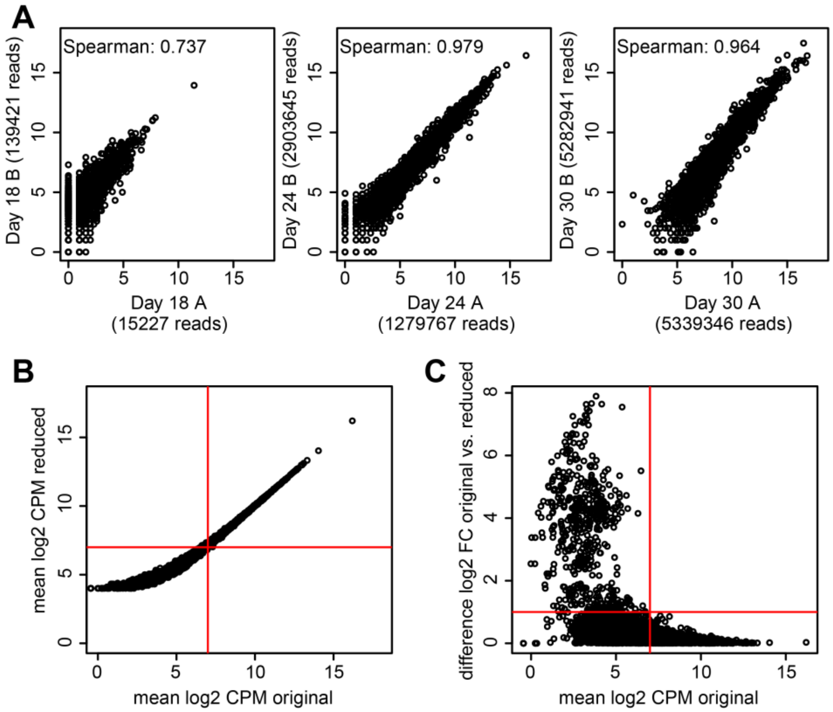

Supplementary Figure S1. Quality control of RNA-seq data. (A) Scatter plots and Spearman rank correlation of the two biological replicates for each day. (B) Reduction of the mapping reads to 100 k leads to underrepresentation of genes with low coverage. (C) Comparison of the calculated $\log_2$ fold changes of the original and the dataset with reduced number of mapping reads reveals huge deviations for genes with a $\log_2$ CPM < 7. CPM = counts of mapping per million reads per gene; FC =fold change.
