# Supplementary material for: Identification of Genetic Modules Mediating the Jekyll and Hyde Interaction of Dinoroseobacter shibae with the Dinoflagellate Prorocentrum minimum
Source: Front Microbiol. 2015 Nov 13;6:1262. doi: 10.3389/fmicb.2015.01262 (PMC4643747; doi:10.3389/fmicb.2015.01262)
Supplement: Supplementary file 4 [file Image_2.PDF]

Supplementary Figure S2

Identification of genetic modules mediating the Jekyll and Hyde interaction of *Dinoroseobacter shibae* with the dinoflagellate *Prorocentrum minimum*

Hui Wang[1§], Jürgen Tomasch[1§], Victoria Michael[2], Sabin Bhuju[3], Michael Jarek[3], Jörn Petersen[2] and Irene Wagner-Döbler[1#]

[§]contributed equally to this work

[#]corresponding author: Irene.Wagner-Doebler@helmholtz-hzi.de

[1]Helmholtz-Centre for Infection Research (HZI), Microbial Communication, Braunschweig, Germany

[2]German Collection of Microorganisms and Cell Cultures (DSMZ), Microbial Ecology and Diversity Research, Braunschweig, Germany

[3]Helmholtz-Centre for Infection Research (HZI), Genome Analytics, Braunschweig, Germany

Running title: Algae – bacteria interactions

Subject category: Microbe-microbe and microbe-host interactions

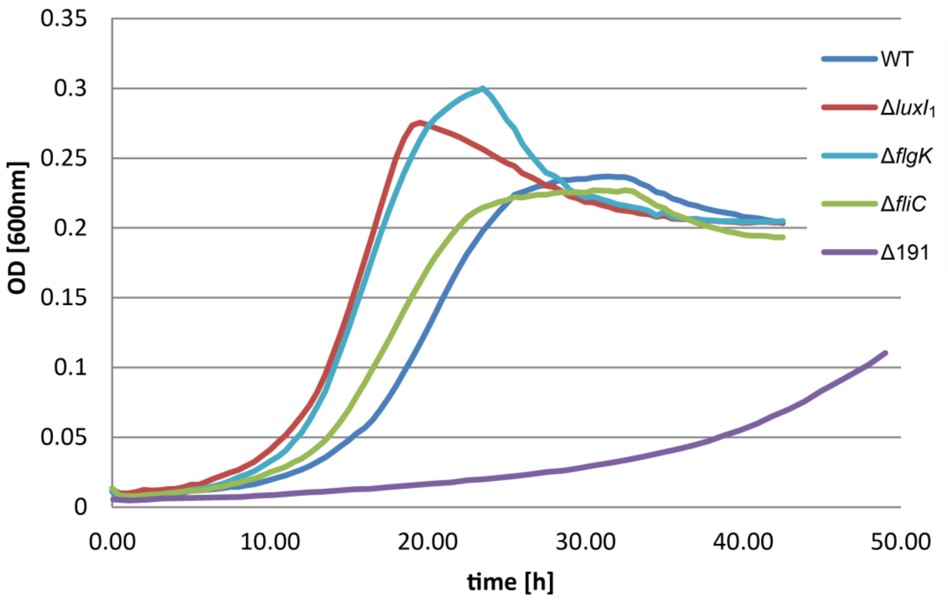

Supplementary Figure S2. Growth of strains used in this study in single culture. The strains were cultivated in artificial seawater medium supplemented with 5 mM succinate as carbon source as described in the material and methods section. The graphs represent the means from 5 to 10 biological replicates. WT: *D. shibae* DFL12 wild-type, OD: optical density at 600 nm.
